# Quantitative Genomics of Aggressive Behavior in Drosophila melanogaster

**DOI:** 10.1371/journal.pgen.0020154

**Published:** 2006-09-15

**Authors:** Alexis C Edwards, Stephanie M Rollmann, Theodore J Morgan, Trudy F. C Mackay

**Affiliations:** Department of Genetics and W. M. Keck Center for Behavioral Biology, North Carolina State University, Raleigh, North Carolina, United States of America; The Jackson Laboratory, United States of America

## Abstract

Aggressive behavior is important for animal survival and reproduction, and excessive aggression is an enormous social and economic burden for human society. Although the role of biogenic amines in modulating aggressive behavior is well characterized, other genetic mechanisms affecting this complex behavior remain elusive. Here, we developed an assay to rapidly quantify aggressive behavior in *Drosophila melanogaster,* and generated replicate selection lines with divergent levels of aggression. The realized heritability of aggressive behavior was approximately 0.10, and the phenotypic response to selection specifically affected aggression. We used whole-genome expression analysis to identify 1,539 probe sets with different expression levels between the selection lines when pooled across replicates, at a false discovery rate of 0.001. We quantified the aggressive behavior of 19 mutations in candidate genes that were generated in a common co-isogenic background, and identified 15 novel genes affecting aggressive behavior. Expression profiling of genetically divergent lines is an effective strategy for identifying genes affecting complex traits.

## Synopsis

Aggressive behavior is a complex trait affected by numerous interacting genes whose expression depends on the environment. Aggression can be selectively advantageous in the pursuit of mates, territory, or food; however, excessive aggression may be deleterious. Pathological levels of aggression in humans create an enormous burden to society. Although dysfunction of the biogenic amine systems is often associated with alterations in aggressive behavior, this represents only the “tip of the iceberg” of the complex genetic architecture of aggressive behavior. The fruit fly Drosophila melanogaster is an excellent model genetic system for exploring the genetic basis of aggressive behavior. The authors have developed a rapid assay to quantify *Drosophila* aggression, and have used it to select genetically divergent replicate lines for increased and decreased behavior from a genetically heterogeneous base population. They used whole-genome expression profiling to identify variation in gene expression among these lines, and identified 1,539 transcripts that differed between the selection lines, illustrating the complex genomic basis of aggressive behavior. The authors evaluated aggressive behavior of flies with mutations in 19 genes that were implicated by the analysis of differential transcript abundance, and identified 15 novel candidate genes affecting this complex trait, eight of which have human orthologs.

## Introduction

Animal aggression is a near-universal survival trait. Aggressive behavior is important for acquisition and defense of food, mates, and progeny; predator avoidance and defense; and, in some animals, the establishment and maintenance of stable social hierarchies. Understanding the genetic, neurobiological, and environmental bases of aggressive behavior is of great importance to human health and society, as this could lead to more effective treatments for the increased levels of aggression observed among patients suffering from many behavioral disorders. Aggressive behavior is a complex, quantitative trait, with population variation attributable to multiple interacting loci with individually small effects, whose expression is contingent on the social and physical environment.

Other than the well-characterized role of biogenic amines in modulating aggressive behavior, little is known of the genetic architecture of this complex trait. In vertebrates, low levels of serotonin (5-hydroxytryptophan [5-HT]) and its metabolites (5-hydroxyindoleacetic acid [5-HIAA]) are associated with increased levels of aggression and impulsivity [1]. Male mice in which the 5-HT_1B_ receptor gene has been ablated are more aggressive than wild type [2]; and mice with a non-functioning 5-HT_1A_ receptor gene are more anxious and less reactive than wild type [3]. These effects are mimicked by pharmacological treatments: increasing levels of 5-HT using 5-HT precursors, 5-HT reuptake inhibitors, and 5-HT_1A_ and 5-HT_1B_ receptor agonists decrease aggressive behavior in rodents [4–8]. Polymorphisms in tryptophan hydroxylase, the rate-limiting enzyme in 5-HT synthesis, affect aggressive disposition in humans [9,10]. 5-HT also mediates aggressive behavior in lobsters and crayfish, but the effects of the serotonergic system in invertebrates are opposite to vertebrates. Crustaceans injected with 5-HT exhibit increased aggression and are less likely to retreat from an aggressive encounter, whereas high levels of octopamine, the invertebrate counterpart of noradrenaline, are associated with subordinate status [11–14].

Monoamine oxidase A (MAOA) oxidizes and degrades 5-HT and dopamine. Inhibition of MAOA activity in mice leads to decreased aggression [15], consistent with increased levels of 5-HT. An allele of MAOA that leads to reduced enzyme activity has been associated with an increase in violent behavior in humans, but only if the individual was abused as a child [16]. In contrast, MAOA deficiency in humans [17] and mice [18] leads to increased aggression, despite the resulting increase in 5-HT levels. Other neurotransmitters and neuromodulators associated with aggressive behavior include nitric oxide (NO) [19,20]; dopamine [21], γ-aminobutyric acid [22], and androgens and estrogens [1].

The role of bioamines is an important focus for studies on aggression, but our current knowledge represents only “the tip of the iceberg” of the complex genetic architecture that subserves aggressive behavior. Hints of this underlying complexity come from studies showing that mice with null mutations in the neural cell adhesion molecule [23], interleukin 6 [24], and the *Nr2e1* nuclear receptor gene [25] are all more aggressive than wild-type litter mates. Further understanding of the genetic basis of aggressive behavior will be greatly facilitated by studies in a genetic model system, such as Drosophila melanogaster. The *Drosophila* biogenic amines have been well characterized [26], and homologous mechanisms may operate in flies and vertebrates, including humans. *Drosophila* males exhibit aggressive behaviors in defense of territory and females [27–31]. Female territorial aggressive behavior has also been quantified [32]. There is substantial naturally occurring genetic variation for levels of aggression, as demonstrated by divergence in behavior among geographical populations [33] and rapid response to artificial selection for increased aggression [34–36]. However, surprisingly little is known about the genes regulating aggressive behavior in *Drosophila,* and none of the loci contributing to naturally occurring variation in aggression have been mapped. However, a handful of genes have mutational effects on aggressive behavior. Mutations in *fruitless* and *dissatisfaction,* two genes involved in the sex determination hierarchy, are associated with increased levels of inter-male aggression [30]. In addition, β-alanine, which can be conjugated to bioamine neurotransmitters, has also been implicated in *Drosophila* aggression. *ebony* mutants have elevated levels of β-alanine and are more aggressive than wild type, whereas *black* mutants have reduced levels of β-alanine and are less aggressive than wild type [28,37]. The neurotransmitters octopamine and dopamine also modulate aggressive behavior in *Drosophila* [37], although the role of serotonin has not been documented.

A major impediment for using *Drosophila* to study the genetic networks underpinning aggressive behavior, and variation in aggressive behavior, has been the lack of a high-throughput assay to quantify this behavior. Most previous studies confounded territorial behavior with mating behavior, and relied on analysis of long-term video recordings to quantify aggressive encounters [31,32]. We developed a rapid and highly reproducible assay to quantify aggressive behavior, and used it to generate replicate lines selected for divergent levels of aggression. We used whole-genome expression analysis to identify candidate genes with different expression levels among the selection lines, an approach that has been fruitful in identifying candidate genes for other behavioral traits [38–40]. Subsequent functional tests of aggressive behavior in lines containing mutations in these candidate genes revealed several novel genes affecting aggressive behavior in *Drosophila*.

## Results

### Direct Phenotypic Response to Selection for Aggressive Behavior

**Figure 1:**
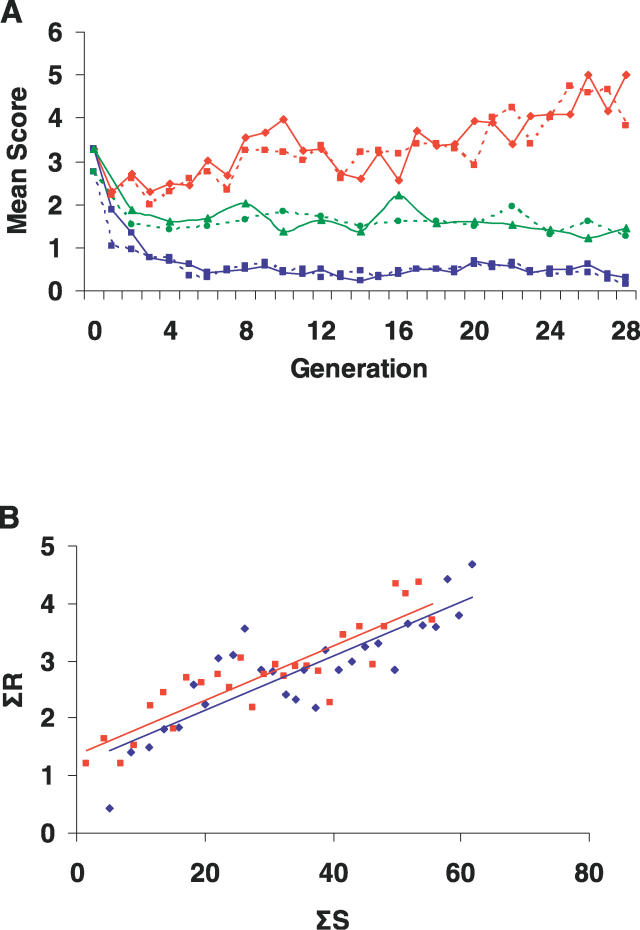
Phenotypic Response to Selection for Aggressive Behavior (A) Mean aggression score of selection lines. Squares () indicate L lines; triangles (▴) indicate C lines; diamonds (♦) indicate H lines; solid lines indicate Replicate 1; and dashed lines indicate Replicate 2. (B) Regressions of cumulative response on cumulative selection differential for divergence between high and low selection lines. Diamonds (♦) and blue line indicate Replicate 1; squares () and red line indicate Replicate 2.

We developed an assay to rapidly measure aggressive behavior of individual *Drosophila*. Briefly, we deprived animals of food for a short period, and then allowed them to compete for and defend a limited food resource. We quantified the aggressive behavior of the focal individual as the total number of aggressive encounters [31] in a 2-min period. We derived a heterogeneous base population from isofemale lines derived from a single natural population, and used artificial selection to create genetically divergent replicate lines with high (H) and low (L) levels of male aggression (Figure 1A). From generation 25–28, the H and L replicate lines diverged by 4.1 aggressive encounters in a 2-min interval, or 3.3 phenotypic standard deviation units.

We estimated realized heritability (*h* ^2^ ± standard error [SE] of the regression coefficient) of aggressive behavior from the regressions of cumulated response on cumulated selection differential [41]. Heritability estimates from the divergence between H and L lines over 28 generations were *h* ^2^ = 0.094 ± 0.0057 (*p* < 0.0001) for Replicate 1 and *h* ^2^ = 0.095 ± 0.0048 (*p* < 0.0001) for Replicate 2 (Figure 1B). The selection response was symmetrical. Estimates of realized heritability were *h^2^ =* 0.098 ± 0.0070 (*p* < 0.0001) and *h^2^ =* 0.107 ± 0.0061 (*p* < 0.0001) for H Replicates 1 and 2, respectively; and *h^2^ =* 0.092 ± 0.0458 (*p* = 0.0018) and *h^2^ =* 0.058 ± 0.0074 (*p* = 0.0006) for L Replicates 1 and 2, respectively. There was little inbreeding depression for aggressive behavior: the regression of aggressive behavior in the control (C) lines over 28 generations was *b* = −0.017 ± 0.0097 (*p* = 0.10) and *b* = −0.033 ± 0.0125 (*p* = 0.02) for C1 and C2, respectively.

### Correlated Phenotypic Response to Selection for Aggressive Behavior

**Figure 2:**
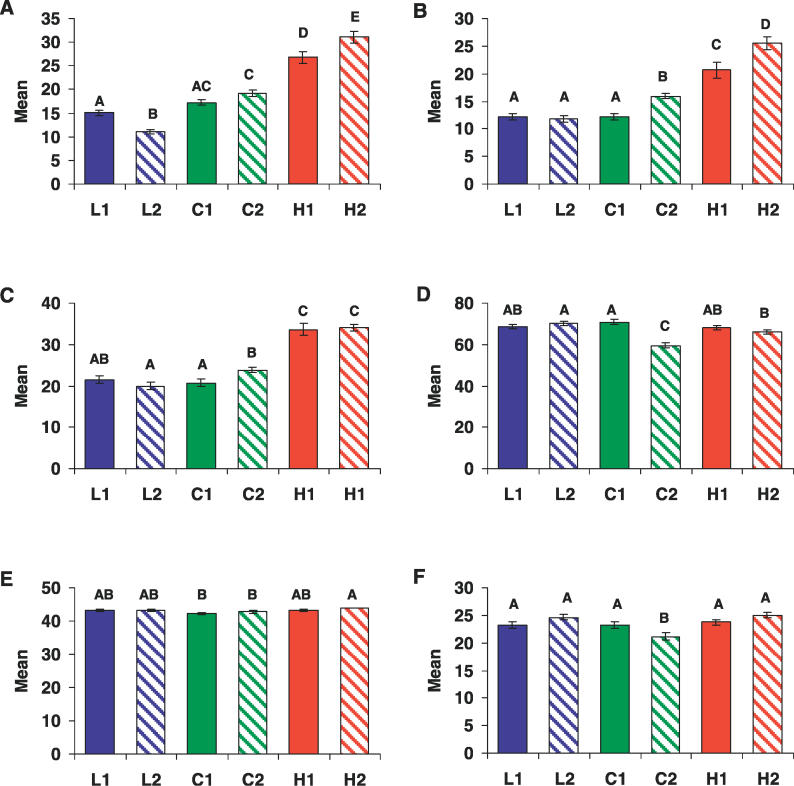
Correlated Phenotypic Responses to Selection All scores are pooled across three successive generations. Lines with the same letter are not significantly different from one another at *p* < 0.05. (A) Male aggression in eight-fly assay. (B) Female aggression in eight-fly assay, after a 90-min food deprivation period. (C) Female aggression in eight-fly assay, after a 120-min food deprivation period. (D) Starvation stress resistance. (E) Locomotor reactivity. (F) Climbing behavior.

*Drosophila* females are typically less aggressive than males [32]. We assessed whether female aggressive behavior changed as a correlated response to selection for divergence in male aggression. Here we used a multiple-fly assay for aggression for three consecutive generations. As expected, there was a significant difference in male aggressive behavior between the selection lines when assessed using this assay (*F*_2,3_ = 19.16, *p* = 0.02, Figure 2A). There was a marginally significant correlated response in female aggressive behavior when females were deprived of food for 90 min (*F*_2,3_ = 11.21, *p* = 0.0405, Figure 2B). However, the correlated response in female aggression was more pronounced after a 2-h deprivation period (*F*_2,3_ = 52.81, *p* = 0.0046, Figure 2C), although the selection group by time interaction term was not significant (*F*_2,3_ = 1.01, *p* = 0.46).

Since we assessed aggressive behavior after a period of starvation, it was important to determine whether the differences in aggressive behavior between the selection lines were not a reflection of underlying differences in sensitivity to starvation stress. There were no significant differences in starvation resistance among the selection lines (*F*_2,3_ = 0.41, *p* = 0.70, Figure 2D). In addition, it was possible that the differences in aggressive behavior were attributable to differences in general locomotion. There were no differences in locomotor behavior among the selection lines using an assay for locomotor reactivity (*F*_2,3_ = 5.47, *p* = 0.10, Figure 2E) and a climbing assay (*F*_2,3_ = 1.97, *p* = 0.28, Figure 2F). Neither were there significant differences among the lines selected for divergent aggressive behavior for mating behavior (*F*_2,3_ = 1.56, *p* = 0.34), cold tolerance (*F*_2,3_ = 0.30, *p* = 0.76), ethanol tolerance (*F*_2,3_ = 0.67, *p* = 0.58), or longevity (*F*_2,3_ = 0.29, *p* = 0.7662) (Figure S1). Thus, the response to selection seems specific for aggressive behavior.

### Transcriptional Response to Selection for Aggressive Behavior

We assessed transcript abundance in the H, L, and C selection lines using Affymetrix high-density oligonucleotide whole-genome microarrays, for flies of the same age and physiological state as selected individuals. We performed these analyses using whole bodies, rather than heads alone, as we wanted to include categories of genes potentially affecting aggressive behavior with more general expression (e.g., genes affecting metabolism and muscle function). Raw expression data are given in Table S1. We used factorial analysis of variance (ANOVA) to quantify statistically significant differences in transcript level for each probe set on the array. Using a false discovery rate [42] of *Q* < 0.001, we found 9,485 probe sets were significant for the main effect of sex, 1,593 were significant for the main effect of line, and 69 were significant for the line × sex interaction (Table S2). All 69 probe sets that were significant for the interaction term were also significant for the main effect of line.

**Figure 3:**
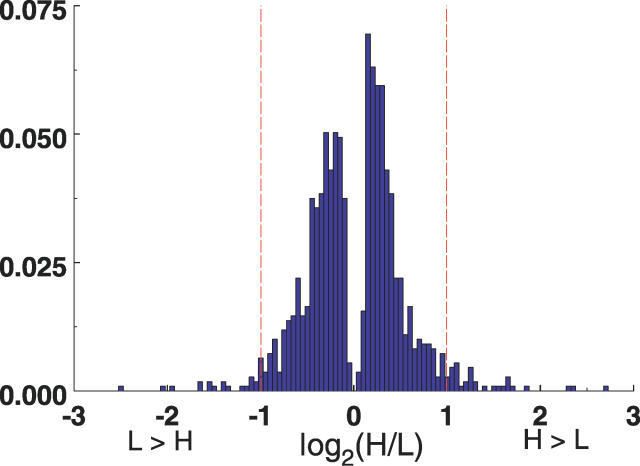
Histogram Showing Frequency of Relative Fold-Change in Probe Sets with Significant Differences in Transcript Abundance between H and L Selection Lines, Pooled over Sexes The vertical red lines demarcate 2-fold changes in transcript abundance.

We used ANOVA contrast statements on the 1,593 probe sets with differences in transcript abundance between selection lines to detect probe sets that were consistently up- or down-regulated in replicate lines [40]. We found 1,539 probe sets (8.2%) that differed between the selection lines when pooled across replicates (Table S3). Most (1,480) of these probe sets were significant in both sexes, consistent with divergence in aggression levels in both sexes. We found 1,116 probe sets that were divergent between H and C, 1,062 probe sets that were divergent between L and C, and 1,083 probe sets that were divergent between H and L. Although there was a widespread transcriptional response to selection for aggressive behavior, the magnitude of the changes of transcript abundance was not great, with the vast majority much less than 2-fold (Figure 3).

We identified 12 transcripts that exhibited sexually antagonistic expression (Table S4). Of these, four differed between the H and L selection lines (*sarcoplasmic calcium-binding protein* was up-regulated in H females and down-regulated in H males; *Transferrin 1, CG8093,* and *CG3239* were up-regulated in H males and down-regulated in H females). *Esterase-10* and *CG4199* had sexually antagonistic effects between the H and C groups (both up-regulated in H females and down-regulated in H males). Finally, six transcripts demonstrated antagonism between the C and L groups (*CG11523* was up-regulated in L males and down-regulated in L females; *twin of eyeless, CG15825, CG8093, CG7598,* and *CG4199* were up-regulated in L females but down-regulated in L males). These probe sets do not share obvious molecular functions or biological processes.

Since we selected for divergence in male aggressive behavior, we tested whether there was a disproportionate contribution of X-linked genes. We assessed whether the 1,539 differentially regulated probe sets were randomly distributed across the five major chromosome arms using a χ^2^ test [40]. Indeed, the distribution of probe sets was not random (χ_4_^2^ =19.66, *p* = 0.0006). However, the deviation from random expectation was attributable to fewer, not more, probe sets than expected on the X chromosome (χ_1_^2^ =13.31, *p* = 0.0003), but not the other chromosome arms (Figure S2). The paucity of probe sets on the X chromosome was due to probe sets that were differentially expressed between males in the contrasts between H and L (χ_1_^2^ = 16.52, *p* = 0.00005), H and C (χ_1_^2^ = 14.53, *p* = 0.00014), and L and C (χ_1_^2^ = 13.49, *p* = 0.00024).

The probe sets with altered transcript abundance between the selection lines fell into all major biological process and molecular function gene ontology (GO) categories (Tables S5 and S6). We used χ^2^ tests to determine which categories were represented more or less frequently than expected by chance, based on representation on the microarray. One interpretation of these analyses is that over-represented GO categories contain probe sets for which transcript abundance has responded to artificial selection, whereas under-represented GO categories contain probe sets for which transcript abundance is under stabilizing natural selection [40]. Highlights of the transcriptional response to artificial selection for aggressive behavior are given in Table 1. For example, the H lines are enriched for up-regulated genes affecting metabolism, response to biotic stimulus, and stress response; whereas the L lines are enriched for up-regulated genes affecting learning and memory and defense response. Probe sets in the biological process categories of morphogenesis and system development are consistently under-represented among up-regulated transcripts in the H and C lines.

**Table 1:**
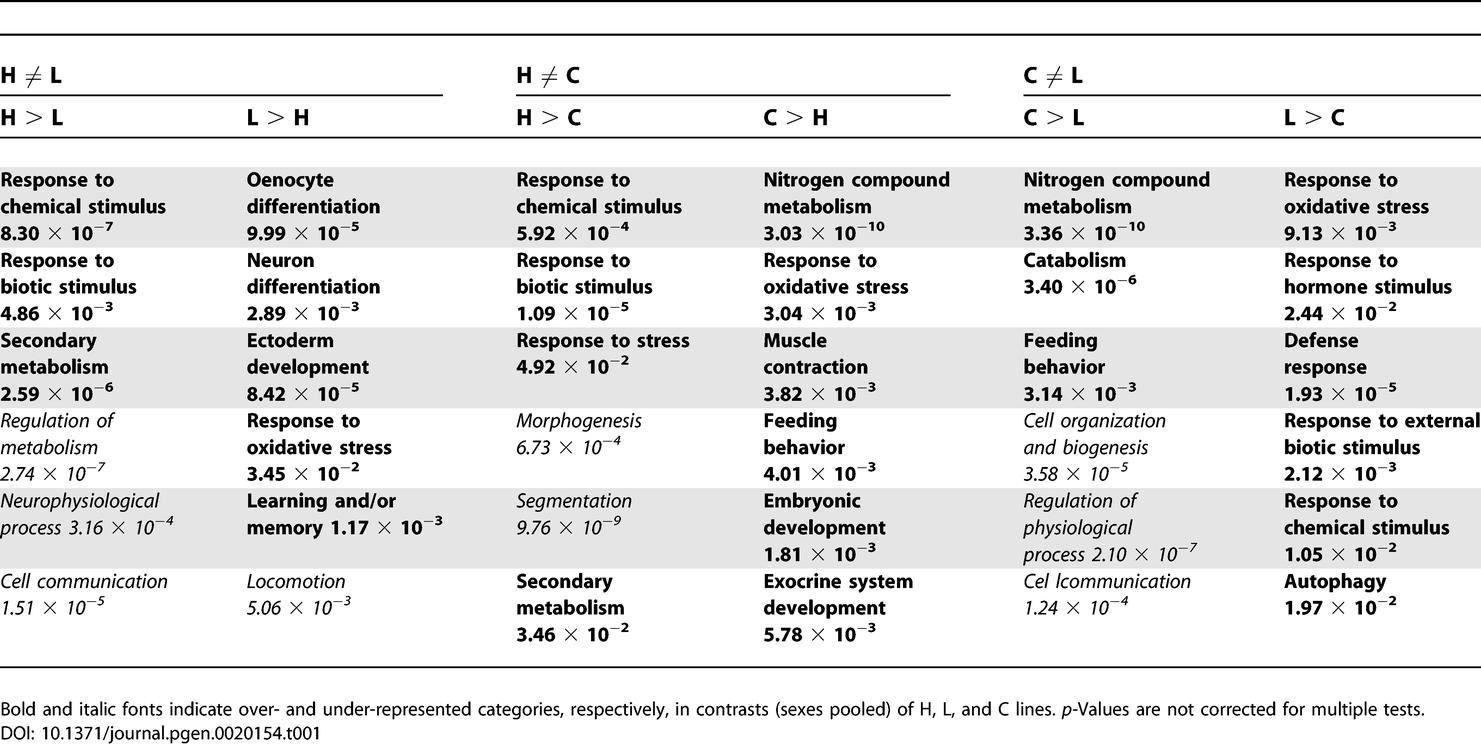
Differentially Represented Biological Process GO Categories

Table 2 gives a sample of candidate genes affecting aggressive behavior that are up-regulated in the high or low selection lines, and which have proven functions in other processes. All are novel candidate genes for aggressive behavior. Conspicuously missing from this list are genes that have been previously implicated in *Drosophila* aggressive behavior *(fruitless, dissatisfaction, ebony,* and *black)* as well as genes affecting bioamines (e.g., genes encoding tyrosine and tyramine hydroxylases; *Dopa decarboxylase;* and dopamine, serotonin, and octopamine; and associated transporters and receptors). Indeed, the only gene affecting bioamine levels that is differentially expressed between the selection lines is Catecholamines up (Table S3).

**Table 2:**
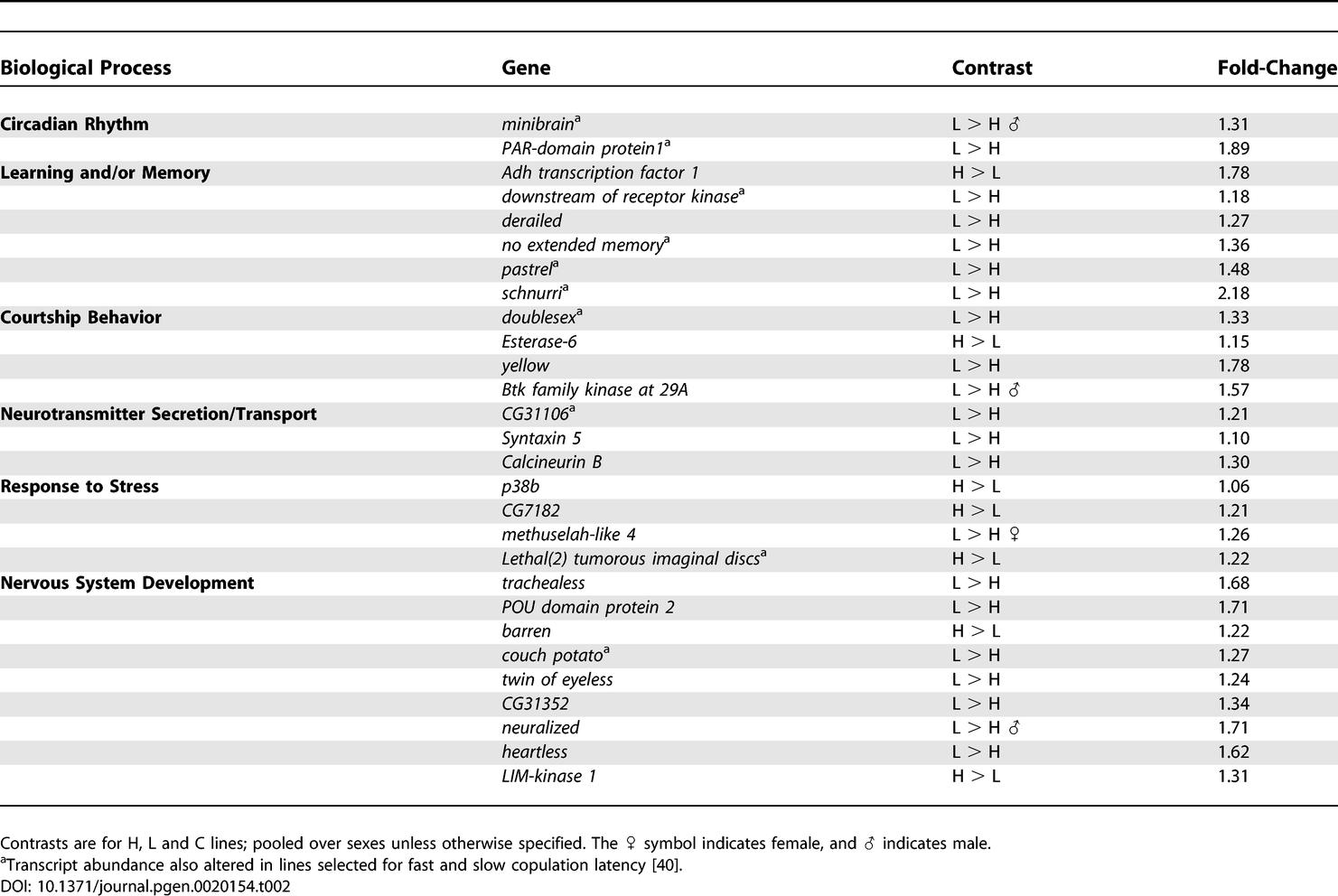
Pleiotropic Candidate Genes Affecting Aggressive Behavior

### Functional Tests of Candidate Genes

**Figure 4:**
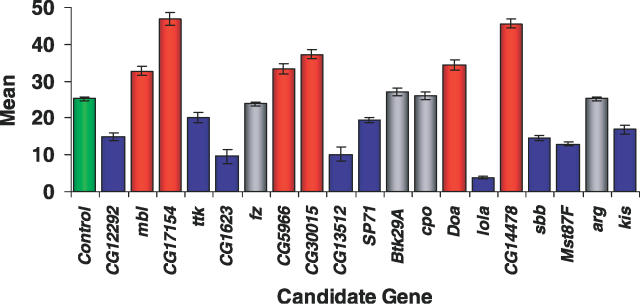
Mean Aggression Scores (±SE) of Lines Containing *P*-Element Insertional Mutations in Candidate Genes The green bar denotes the Canton S B co-isogenic control line; grey bars indicate lines with scores not significantly different from the control; red bars indicate lines with significantly higher levels of aggression than the control; and blue bars indicate lines with significantly lower levels of aggression than the control.

To assess the extent to which transcript profiling of divergent selection lines accurately predicts genes that directly affect the selected trait, we evaluated the aggressive behavior of lines containing *P*-element insertional mutations in 19 candidate genes that were implicated by the analysis of differential transcript abundance. All of the *P*-element insertions were derived in a common isogenic background, and are viable and fertile as homozygotes [43,44]. The *P*-elements are inserted in, or immediately adjacent to, each candidate gene. The candidate genes are involved in diverse biological processes, including electron transport *(Male-specific RNA 87F)*, catabolism *(arginase)*, nervous system development *(longitudinals lacking, tramtrack,* and *muscleblind)*, and G-protein coupled receptor signaling *(frizzled)*. Seven of the mutations are in computationally predicted genes *(CG1623, CG5966, CG12292, CG13512, CG14478, CG17154,* and *CG30015)*. Remarkably, 15 of the mutations exhibited significant differences in aggressive behavior from the co-isogenic control line, after Bonferroni correction for multiple tests (Table 3, Figure 4). Mutations in *muscleblind, CG17154, CG5966, CG30015, Darkener of apricot,* and *CG14478* had higher levels of aggression than the control, and mutations in *CG12292, tramtrack, CG1623, CG13512, SP71, longitudinals lacking, scribbler, Male-specific RNA 87F,* and *kismet* were less aggressive than the control. None of these genes have been previously implicated to affect aggressive behavior.

**Table 3:**
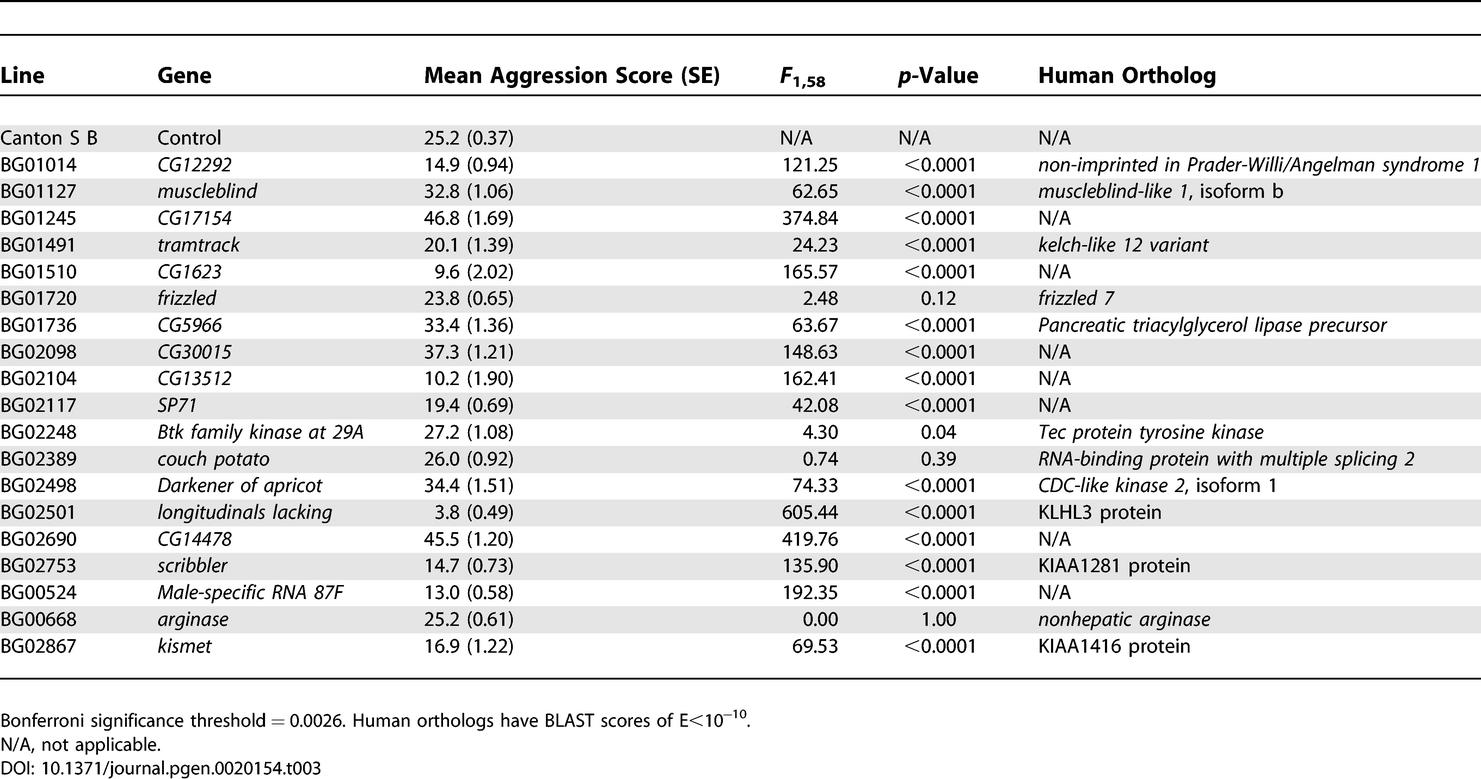
Functional Tests of Candidate Genes

## Discussion

### Phenotypic Response to Selection for Aggressive Behavior

Drosophila melanogaster exhibits a robust response to artificial selection for high and low levels of aggressive behavior. The heritability of aggressive behavior is relatively low (~0.1). However, if we express the genetic and environmental variances of aggressive behavior as genetic and environmental coefficients of variation (*CV_G_* and *CV_E_,* respectively [45]), we find that *CV_G_* = 23.2 and *CV_E_* = 71.9. Thus, the low heritability is not due to a lack of segregating genetic variation, which is abundant, but to a high level of environmental variance, as is typical for behavioral traits [45]. Although the phenotypic response to artificial selection appears to be greater in the direction of increased levels of aggression, the genetic response to selection as inferred from realized heritability is symmetrical. The apparent discrepancy is attributable to the low phenotypic variance, and hence selection differential, in the L lines. The symmetrical selection response is consistent with natural selection for an intermediate optimum level of aggression, since fitness traits under directional natural selection typically exhibit asymmetrical responses to artificial selection, in the direction of reduced fitness [46]. This is consistent with the intuitive notion that both highly aggressive and very passive individuals would be at a selective disadvantage. Time spent in aggressive interactions cannot be at the expense of locating food and mates; further, aggressive behavior is energetically expensive, and must be limited accordingly.

The phenotypic response to selection appears to be specific for aggressive behavior. In particular, the differences in aggressive behavior among the selection lines are not due to general differences in activity or differential sensitivity to starvation stress. The lack of correlation with cold tolerance and longevity also indicates that the selection response is not directly related to differences in general stress response or physiological robustness. Neither do increased or decreased levels of aggression in the context of a limited food resource affect mating behavior, as measured by copulation latency. The latter observation is not necessarily at variance with previous reports that males with increased levels of territorial aggression appear to have a mating advantage [27,36]. The correlated response of copulation latency to selection for aggression is expected to be *i h_CL_ h_AG_ r_A_ σ_PCL_,* where *i* is the selection intensity; *h_CL_* and *h_AG_* are the square roots of the heritabilities of copulation latency and aggression, respectively; *r_A_* is the genetic correlation between the two traits; and *σ_PCL_* is the phenotypic standard deviation of copulation latency [41]. From a previous study of response to selection for copulation latency from the same base population, we have estimates of *h* ^2^ *_CL_* = 0.067 and *σ_PCL_* = 18.7, whereas this study gives estimates of *h* ^2^ *_AG_* = 0.0945 and *i* = 0.951. Thus, after 28 generations of selection for divergent aggressive behavior, we would expect a correlated response in copulation latency of 39.62*r_A_* min. We would only have the power to detect this correlated response if *r_A_* was very high, but not if *r_A_* < 0.3.

### Transcriptional Response to Selection for Aggressive Behavior

We observe a profound transcriptional response to selection for aggressive behavior, with changes in expression of over 1,500 probe sets (~10% of the genome) between the selection lines, using a stringent false discovery rate of 0.001. Similarly, transcript abundance of over 3,700 probe sets evolved as a correlated response to artificial selection from the same base population for increased and decreased copulation latency [40]. This is in contrast to an analysis of transcriptional response to selection for geotaxis behavior [38] in which 5% of the genes assessed exhibited 2-fold or greater differences in expression between the selection lines. The discrepancy is likely attributable to the different methods for identifying differentially regulated transcripts. We find that transcripts exhibiting much less than 2-fold differences are often highly statistically significant.

We selected for divergence in male aggressive behavior, and therefore expected an increased selection response from X-linked genes affecting variation in male aggression. In contrast, at the level of transcript abundance, there were fewer than expected male-specific differences in expression between the selection lines for X-linked genes. Under-representation of genes that are up-regulated in males on the *Drosophila* X chromosome is apparently a general phenomenon [40,47,48]. X chromosome demasculinization is perhaps attributable to selection against genes that are advantageous in males but deleterious to females [47].

The large number of genes exhibiting parallel changes in transcript abundance among replicate selection lines implies that genes affecting complex behaviors are highly pleiotropic: if 10% of the genome affects any one trait, the same genes must affect multiple traits. Thus, genes affecting behavior are also likely to be involved in neurogenesis, metabolism, development, and general cellular processes, and many of the same genes may affect multiple behaviors. In a previous study, we observed a total of 3,727 probe sets that were differentially expressed between lines selected for increased and decreased copulation latency, from the same base population. A total of 878 probe sets with different expression levels between selection lines were common between lines selected for divergent mating behavior and aggression, which is significantly more overlap than expected by chance (χ^2^ = 1,072.108, *p* < 0.0001). For example, *Pigment dispersing factor* and *cryptochrome* were initially defined based on their involvement in circadian rhythm. Expression levels of these genes were up-regulated in lines selected for positive geotaxis, and confirmed to affect geotaxis behavior in functional tests [38]. *Pigment dispersing factor* and *cryptochrome* were also differentially expressed between the lines selected for increased and decreased mating speed, and here between lines selected for different levels of aggressive behavior (Table S3).

The dual observations of specific responses to artificial selection at the level of trait phenotype and large scale pleiotropy at the level of transcript abundance are not incongruent. Correlated responses to selection can only occur if the genetic correlation between the selected and the correlated trait is non-zero. Significant genetic correlations result from linkage and from net directional pleiotropic effects of genes affecting both traits [41]. We speculate therefore that pleiotropic genes affecting multiple complex traits may not be directional. Further, genetic correlations arise from the segregation of polymorphic alleles affecting both traits. The transcriptional response to selection is attributable to genes that have causally responded to selection, and to genes that are co-regulated by these genes. Since the transcriptional response to single mutations with subtle phenotypic effects can involve over 100 co-regulated genes [36], the number of selected loci causing the changes in transcript abundance between the selection lines could well be rather modest. It will be necessary to map the quantitative trait loci (QTLs) causing divergence between the selection lines in order to disentangle causal versus consequential transcriptional responses and correlated responses to selection.

### Candidate Genes for Aggressive Behavior

Regardless of whether or not the observed changes in genes expression are causally associated with genetic divergence in aggression between the selection lines, the genes exhibiting altered expression levels are candidate genes affecting aggressive behavior. We tested for aggression levels of 19 mutations in candidate genes that were generated in a common co-isogenic background, and identified 15 novel genes affecting aggressive behavior. Aggressive behavior is the first annotated biological function for seven of these mutations, which were in computationally predicted genes. *Male-specific RNA 87F* is involved in electron transporter activity and iron ion binding. The energetic costs of aggression are presumably high, and the mutant tested exhibited low aggression, suggesting that disruption of normal energetic processes adversely affects the ability to engage in costly behaviors. *muscleblind* encodes a protein with a zinc-finger domain involved in muscle development. Proper muscle development could directly affect the frequency and/or intensity of aggressive interactions, which can involve tussling or other elaborate postures. The remaining mutations with effects on aggressive behavior *(tramtrack, Darkener of apricot, longitudinals lacking,* and *scribbler)* were in genes affecting nervous system development. The expression of aggression requires integration of environmental and internal signals for effective behavioral output. Both afferent and efferent signaling can be perturbed by changes in neural activity or functioning; although the effects of these mutations on the neural circuitry involved in aggression remains to be elucidated. Further analyses are required to formally prove the involvement of these genes in aggression, including but not limited to generating multiple mutant alleles, assessment of temporal and spatial patterns of gene expression, transgene rescue, and evaluation of aggressive behavior in animals in which the genes are over-expressed or reduced.

The high success rate of these functional tests validates using expression profiling on genetically divergent lines in directed mutagenesis screens to identify genes affecting complex traits. This strategy is complementary to traditional strategies and cannot supplant them, since many key genes will not be detected as differentially expressed. Specifically, we will not detect genes that are differentially expressed at a different developmental period or if the magnitude of the difference in transcript abundance is too small to be detected; genes that affect protein abundance or activity; or genes affecting the trait that are not regulated at the level of transcription. Notably, we did not detect differences in transcript abundance between the selection lines for genes for which mutations are known to affect aggressive behavior. This observation highlights the difference between the complementary approaches of forward genetic screens and assessing natural variants for inferring the genetic architecture of complex behaviors. The former approach is invaluable for determining the full spectrum of genes affecting the manifestation of behavior, whereas the latter focuses on the subset of genes in which variants have survived the sieve of natural selection. Thus, mutations in genes that were previously determined to affect aggressive behavior may be too deleterious to remain segregating in nature.

Many of the genes with mutational effects on aggressive behavior are evolutionarily conserved and have human orthologs (Table 3). For example, *CG12292* is orthologous to *nonimprinted gene in prader-willi syndrome/angelman syndrome chromosome region 1*. Prader-Willi/Angelman syndrome is a developmental disorder in which many affected individuals exhibit dramatic behavioral phenotypes. It is thus possible that the genes and pathways affecting aggression in *Drosophila* will elucidate corresponding mechanisms in other organisms, including humans.

## Materials and Methods

### Drosophila stocks.

Flies were reared on cornmeal/molasses/agar medium under standard culture conditions (25 °C, 12:12 h light/dark cycle). CO_2_ was used as an anesthetic. Behavioral assays were conducted in a behavioral chamber (25 °C, 70% humidity) between 8 a.m. and 11 a.m.

### Quantitative assay for individual aggressive behavior.

Behavioral assays were performed on socially experienced, 3–7-d-old males. Flies were not exposed to anesthesia for at least 24 h prior to the assay. Aggression of single individuals was quantified by placing one experimental male, with wild-type eye color, with three reference white-eyed isogenic *w* ^1118^ Canton S males. The flies were placed in a vial without food for 90 min, after which they were transferred (without anesthesia) to a test arena containing a droplet of food and allowed to acclimate for 2 min. After the acclimation period, the flies were observed for 2 min. The following behaviors were scored as aggressive encounters: kick—leg extension from one fly to another resulting in physical contact; chase; charge—rapid approach leading to head-to-head orientation; wing-raise—extension of wings in response to proximity/approach of another fly; and box—high impact interaction involving front legs of both flies [31]. The score of the experimental fly was the number of encounters in which it exhibited an aggressive behavior, including interactions initiated by the experimental fly and those in which he responded aggressively to a reference fly.

### Artificial selection for aggressive behavior.

The base population was generated from 60 isofemale lines established from flies collected in Raleigh, North Carolina, in 1999. The isofemale lines were crossed in a round robin design (Line 1 female × Line 2 male, Line 2 female × Line 3 male,...Line 60 female × Line 1 male). Single fertilized females from each cross were placed in each of two culture bottles. In the following generation (G0), the aggressive behavior of 50 virgin males of each replicate was scored using the single-fly assay. The 20 most aggressive males from each replicate were placed with 20 unselected virgin females in bottles to initiate the two H lines (H1 and H2); and the 20 least aggressive males from each replicate were placed with 20 unselected virgin females to initiate the two L lines (L1 and L2). The two C lines were initiated with 20 random, unselected males mated with 20 virgin females. In the following (G1) and all subsequent generations, the same procedure was repeated: 50 males from each line (H, L, and C) were scored, and the 20 highest-scoring males from the H lines and the 20 lowest-scoring males from the L lines were selected as parents for the next generation. The first 20 C line males scored were used as parents. C lines were scored every other generation.

Estimates of realized heritability (*h* ^2^) were calculated by regression of the cumulative selection response (Σ*R*) on the cumulative selection differential (Σ*S*) [41]. The coefficients of genetic *(CV_G_)* and environmental *(CV_E_)* variation were calculated as *CV_G_* = 100(√*V_G_*)/ x̄ and *CV_E_* = 100(√*V_E_*)/ x̄. *V_G_* was estimated as *h* ^2^ *V_P_,* where *V_P_* was the average phenotypic variance of the control lines in generations 1–10. *V_E_* was estimated as *V_P_* − *V_G_*. The mean ( x̄) was estimated as the mean aggression score of the control lines from generations 1–10.

### Correlated responses to selection.

To assess the generality of the selection response, we also assessed male aggression levels in groups of eight 3–7-d-old flies of the same genotype. The aggression score for each replicate was the total number of aggressive interactions observed among all eight flies in the 2-min observation period. We also examined correlated responses in female aggression using the eight-fly assay.

We assessed female aggression after 90-min and 120-min starvation periods. These assays were performed on ten replicates per line per sex for each of three generations; males were assessed at generations 19–21; females were assessed at generations 20–22 (90-min food deprivation) and generations 23–35 (120-min food deprivation).

Starvation resistance was assessed as previously described [49]. Single-sex groups of ten 2-d-old flies were placed in vials containing non-nutritive media (1.5% agar and 5-ml water). Survival was scored every 8 h. This assay was conducted for generations 24–26, with five replicate measurements per line per sex per generation.

Locomotor behavior was assessed using two different assays. Locomotor reactivity was assessed as described previously [50]. A single 3–7-d-old fly was placed in a vial with approximately 3-ml standard medium, and subjected to gentle mechanical disturbance by tapping on the bench top. The vial was placed horizontally, and the total amount of time (in seconds) the fly remained mobile for the 45-s period immediately following the disturbance was the locomotor reactivity score of the individual. This assay was performed at generations 23–25, with 20 replicate measurements per line per sex per generation. In the second assay, individual flies were transferred without anesthesia into an empty glass vial, with the height of the vial demarcated in 5-mm intervals from 0 to 27. The fly was tapped to the bottom of the vial, which was then placed vertically. The climbing score was the maximum height reached within the 10-s observation period. Twenty replicates per line per sex were tested at generations 24–46.

Chill-coma recovery was quantified as previously described [51]. Twenty-five 3–7-d-old flies were transferred without anesthesia into an empty vial and placed on ice for 3 h. The flies were then transferred to room temperature, and the recovery time was recorded as the length of time necessary for an individual to right itself and stand on its legs. The assay was performed at generations 26–28.

Ethanol sensitivity was measured using an inebriometer [52]. Briefly, approximately50–60 same-sex flies were aspirated into a glass column with mesh partitions, which was filled with saturated ethanol vapors. The flies lose postural control due to ethanol exposure and fall down the partitions to the bottom of the column where they were collected at 1-min intervals. The elution time was recorded as the measure of ethanol sensitivity. This assay was conducted for generations 24–26.

Longevity of mated males and females was quantified as previously described [53]. Three male and three female 2-d-old flies were placed in a vial containing approximately 3-ml standard culture medium, and scored for survival every other day until all were dead. Animals were transferred to fresh vials every 2 d. This assay was performed at generations 21–23, with ten replicate vials per line per generation.

Copulation latency was scored as previously described [40]. For each selection line, 20 pairs of 3–7-d-old virgin flies were aspirated into vials containing approximately 3-ml standard culture medium. The score recorded for a pair was the number of minutes from introduction to the vial until initiation of copulation. Reciprocal matings (females from the low selection group mated to males from the high selection group, and vice versa) were also performed. Assays were performed at generations 24–26.

### Statistical analysis of correlated responses.

Differences between the selection lines for the correlated traits were assessed using a nested mixed model ANOVA:

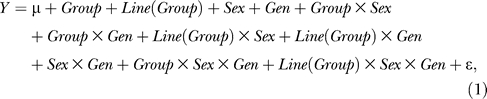
where *Y* is the phenotypic score, *μ* is the overall mean, *Group* is the fixed effect of the selection treatment (H, C, or L), *Line*(*Group*) is the random effect of the replicate within each selection group, *Sex* is the fixed effect of sex, *Gen* is the fixed effect of generation, and *ɛ* is the error variance. The terms of most interest in the model are *Group, Line*(*Group*), *Group×Sex,* and *Line*(*Group*)*×Sex*. A significant *Group* term is indicative of a correlated response in the trait being tested to selection for aggressive behavior. The *Line*(*Group*) term reveals whether replicate lines responded similarly or divergently, giving an idea of the effects of random genetic drift within a replicate line. Interaction terms including the main effect of *Sex* are of interest in part because only males were directly subjected to selection.

### Whole-genome expression profiling.

At generation 25, two replicates of 12 3–7-d-old virgin males and females were collected from each selection line, and deprived of food for 90 min (i.e., the same age and physiological state as the flies prior to selection). Total RNA was extracted from the 24 samples (six lines × two sexes × two replicates) using the Trizol reagent (Gibco BRL, San Diego, California, United States). Biotinylated cRNA probes were hybridized to high-density oligonucleotide microarrays (Drosophila GeneChip 2.0; Affymetrix, Santa Clara, California, United States) and visualized with a streptavidin–phycoerythrin conjugate, as described in the Affymetrix GeneChip Expression Analysis Technical Manual (2000), using internal references for quantification. The quantitative estimate of expression of each probe set is the *Signal (Sig)* metric, as described in the Affymetrix Microarray Suite, version 5.0.

### Microarray data analysis.

The 18,800 probe sets on the Affymetrix Drosophila GeneChip 2.0 are represented by 14 perfect-match (PM) and 14 mismatch (MM) pairs. The *Sig* metric is computed using the weighted log(PM–MM) intensity for each probe set, and was scaled to a median intensity of 500. A detection call of Present, Absent, or Marginal is also reported for each probe set. We excluded probe sets with more than half of the samples called “Absent” from the analysis, leaving 11,666 probe sets. This filter retained sex-specific transcripts, but eliminated probe sets with very low and/or variable expression levels [40]. On the remaining probe sets, we conducted two-way fixed effect ANOVAs of the Signal metric, using the following model:

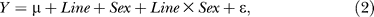
where *Sex* and *Line* are the fixed effects of sex and selection line, and *ɛ* is the variance between replicate arrays. We corrected the *p-*values computed in these ANOVAs for multiple tests using a stringent false discovery rate criterion [42] of *Q* < 0.001. We used contrast statements [40] to assess whether expression levels of probe sets with *Line* and/or *Line*×*Sex* terms at or below the *Q* = 0.001 threshold were significantly different between selection groups (H, C, and L) at the *p* < 0.05 level, both within each sex and pooled across sexes. GO categories were annotated using Affymetrix (http://www.affymetrix.com) and FlyBase (http://flybase.bio.indiana.edu) compilations.

### Functional tests of mutations in candidate genes.

We tested whether mutations in 19 of the candidate genes with altered transcript abundance between the selection lines affected aggressive behavior. The mutations were homozygous *P{GT1}* elements inserted within the candidate genes, and all were generated in a common co-isogenic background (Canton S, B background [44]). Male aggressive behavior was assessed for all mutant lines using the eight-fly assay, with ten replicates per line, and for 50 replicates of the co-isogenic control line (Canton S, B background). We used *t*-tests to determine whether the aggressive behavior of the mutant lines differed significantly from the control.

## Supporting Information

Figure S1 Correlated Phenotypic Responses to Selection (32 KB DOC)

Figure S2 Genomic Distribution of Probe Sets (40 KB DOC)

Table S1 Raw Microarray Data (4.8 MB ZIP)

Table S2 Probe Sets Differing Significantly by Selection Line Term (847 KB XLS)

Table S3 Probe Sets with Significant Differences in Contrast Statements (414 KB XLS)

Table S4 Sexually Antagonistic Probe Sets (19 KB XLS)

Table S5 Biological Process GO Categories (521 KB XLS)

Table S6 Molecular Function GO Categories (290 KB XLS)

### Accession Numbers

Raw microarray data have been deposited in Gene Expression Omnibus (GEO) (http://www.ncbi.nlm.nih.gov/geo) under accession number GSE5405.

## Abbreviations

ANOVA: analysis of variance
C: control
GO: gene ontology
H: high aggression
L: low aggression
MAOA: monoamine oxidase A
SE: standard error
5-HT: 5-hydroxytryptophan
