# Supplementary material for: Quantitative Genomics of Aggressive Behavior in Drosophila melanogaster
Source: PLoS Genet. 2006 Sep 15;2(9):e154. doi: 10.1371/journal.pgen.0020154 (PMC1564424; doi:10.1371/journal.pgen.0020154)
Supplement: Supplementary file 1 [file pgen.0020154.sg001.doc]

**Supplementary Figure 1.** Correlated phenotypic responses to selection.

All scores are pooled across three successive generations. Lines with the same letter are not significantly different from one another at *P* < 0.05. L: Low aggression lines; H: High aggression lines; C; Control lines. 1 and 2 denote independent replicates.
